# Reverse cannulation and dilation successfully assist the cannulation of the accessory pancreatic duct

**DOI:** 10.1055/a-2462-1825

**Published:** 2024-11-26

**Authors:** Yi-Li Cai, Fan Wang, Miao Liu, Ting Yang, Zhao-Shen Li, Liang-Hao Hu

**Affiliations:** 1 Department of Gastroenterology, Changhai Hospital, Naval Medical University, Shanghai, China; 2 Digestive Endoscopy Center, Changhai Hospital, Naval Medical University, Shanghai, China

A 68-year-old woman with chronic pancreatitis and pancreatic duct stones was admitted to hospital due to recurrent abdominal pain. Extracorporeal shock wave lithotripsy was performed before endoscopic retrograde cholangiopancreatography (ERCP). Pancreatogram revealed that the main pancreatic duct (MPD) was twisted and formed an α loop structure, which made stent placement impossible ( Fig. 1 ). In this case, the accessory pancreatic duct (APD) assumed the drainage function.

**Fig. 1:**
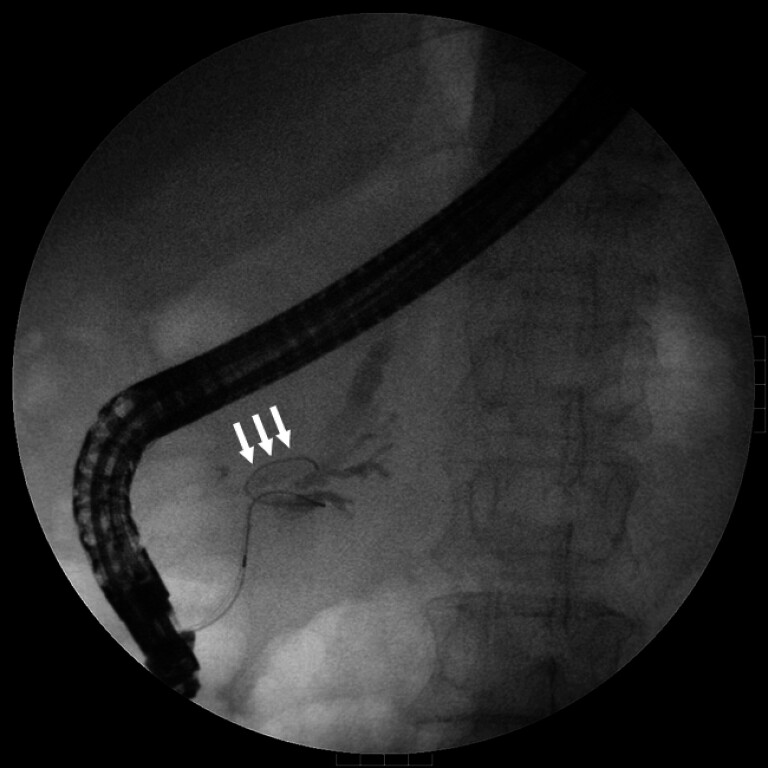
Pancreatogram revealed that the main pancreatic duct was twisted and formed an α loop structure (white arrows).

The operator made several attempts to cannulate the minor papilla, all of which were unsuccessful due to the inconspicuous minor papilla orifice. Eventually, the guidewire (450 cm, Acrobat; Wilson-Cook Medical Inc., Winston Salem, North Carolina, USA) in the MPD successfully passed through the minor papilla and coiled in the duodenal lumen ( Fig. 2 **a, b** ). Then, a 6-Fr bougie and a 7-Fr bougie were sequentially used to dilate the APD and minor papilla from the inside out ( Fig. 2 **c, d** ). Sphincterotomy was performed on the minor papilla using a DualKnife (Olympus Corp., Tokyo, Japan). A new guidewire (450 cm, Jagwire; Boston Scientific Corp., Marlborough, Massachusetts, USA) was then placed in the APD. Pancreatogram showed that the morphology of the APD was suitable for stent placement ( Fig. 3 **a** ). After dilation of the APD with a Hurricane balloon (Boston Scientific Corp.), the remaining stones in the pancreatic duct were removed using a balloon and a basket ( Fig. 3 **b, c, d** ). A plastic stent (8.5 Fr, 5 cm) was then placed in the pancreatic duct. Pancreatic juice was seen flowing out of the stent ( Video 1 ).

**Fig. 2:**
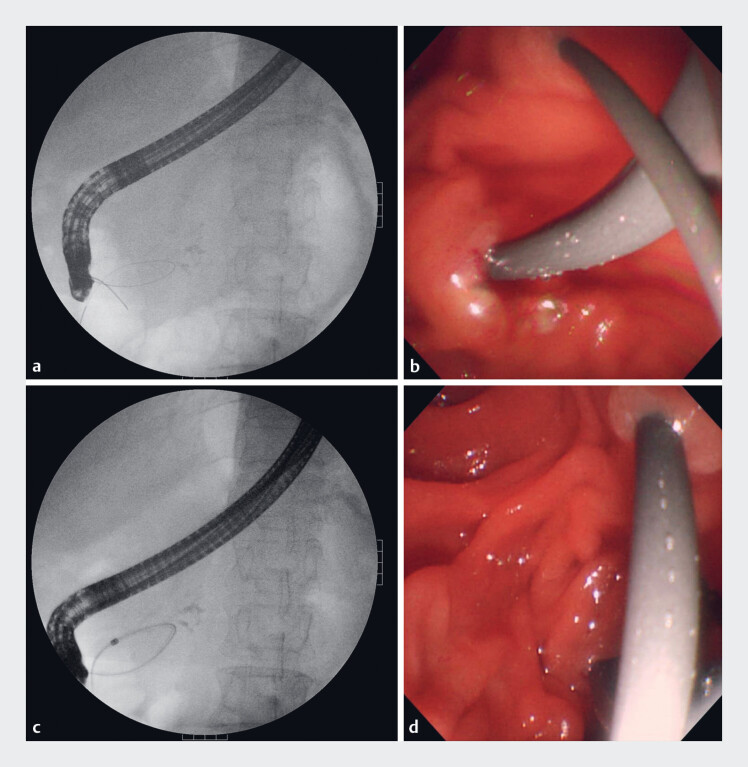
Guidewire and bougie placement. **a, b** The guidewire in the main pancreatic duct passed through the minor papilla and coiled in the duodenal lumen. **c, d** A bougie was sent along the guidewire and passed through the minor papilla.

**Fig. 3:**
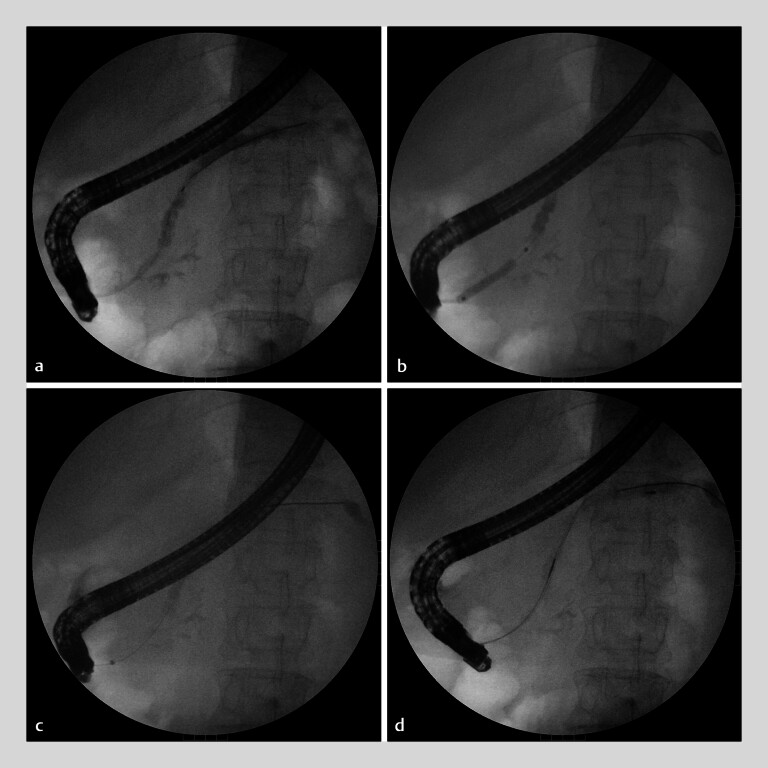
Stone clearance. **a** Pancreatogram showed the morphology of the accessory pancreatic duct. **b** A Hurricane balloon (Boston Scientific Corp., Marlborough, Massachusetts, USA) was used to dilate the accessory pancreatic duct. **c, d** The remaining stones in the pancreatic duct were cleared using a balloon ( **c** ) and a basket ( **d** ).

Reverse cannulation and dilation aided stent placement in the accessory pancreatic duct.Video 1

Placing a pancreatic stent under ERCP is the first-line treatment for chronic pancreatitis with pancreatic duct stenosis 1 . This study proposed a new method, referred to as the reverse cannulation/dilation technique, of assisting the cannulation and dilation of the minor papilla in patients with chronic pancreatitis and a strongly twisted MPD, in whom direct cannulation of the minor papilla is difficult.

Endoscopy_UCTN_Code_TTT_1AR_2AH

## Notes

Endoscopy E-Videos https://eref.thieme.de/e-videos
*E-Videos* is an open access online section of the journal *Endoscopy* , reporting on interesting cases and new techniques in gastroenterological endoscopy. All papers include a high-quality video and are published with a Creative Commons CC-BY license. Endoscopy E-Videos qualify for HINARI discounts and waivers and eligibility is automatically checked during the submission process. We grant 100% waivers to articles whose corresponding authors are based in Group A countries and 50% waivers to those who are based in Group B countries as classified by Research4Life (see: https://www.research4life.org/access/eligibility/ ). This section has its own submission website at https://mc.manuscriptcentral.com/e-videos .
